# The Correlation between Osteoporosis Occurrences in Both Schizophrenia and Parkinson’s Disease

**DOI:** 10.3389/fneur.2014.00083

**Published:** 2014-06-02

**Authors:** Fatemeh Radaei, Asma Darvishi, Shahriar Gharibzadeh

**Affiliations:** ^1^Neural and Cognitive Sciences Laboratory, Biomedical Engineering Faculty, Amirkabir University of Technology, Tehran, Iran; ^2^Tehran University of Medical Sciences, Tehran, Iran

**Keywords:** Parkinson’s disease, schizophrenia, osteoporosis, AMPK, energy metabolism

Osteoporosis disease is a metabolic disorder in which bone mineral density (BMD) is lower than the normal threshold. Based on literature, it is known that schizophrenic patients due to consuming anti-psychotic drugs and Parkinson’s disease patients due to vitamin D deficiency and decrease in mobility are at the high risk of osteoporosis (1–4).

On the other hand, it is observed that adenosine 5′-monophosphate (AMP)-activated protein kinase (AMPK) activity, which is regulating cellular energy homeostasis, is reduced in schizophrenia diseases (5). In addition, it is known that there is mitochondrial dysfunction in Parkinson’s disease that can be treated by AMPK, which is identified as a mitochondrial biogenesis (6–8). Recently, some findings show that AMPK plays an important role in bone metabolism. Besides, some *in vitro* studies revealing that AMPK modulators regulate bone cell function (9–13). Also, some studies show that deficiency of AMPK α and β subunits in mice causes bone loss *in vivo* (14).

Based on the above mentioned points, it can be referred that AMPK deficiency in Parkinson’s disease and schizophrenia may lead to osteoporosis. This can be used as a goal in treatment of osteoporosis in those disorders. In another word, nowadays there are some drugs available for both diseases but they have some side effect, which may lead to osteoporosis; by considering the fact that AMPK deficiency may cause osteoporosis, new drugs can be provided with AMPK supplement to reduce the osteoporosis symptoms. Surely, experimental trials are needed to validate our hypothesis.

## Conflict of Interest Statement

The authors declare that the research was conducted in the absence of any commercial or financial relationships that could be construed as a potential conflict of interest.
